# The Polyamine Spermine Potentiates the Propagation of Negatively Charged Molecules through the Astrocytic Syncytium

**DOI:** 10.3390/biom12121812

**Published:** 2022-12-05

**Authors:** Jan Benedikt, Christian J. Malpica-Nieves, Yomarie Rivera, Miguel Méndez-González, Colin G. Nichols, Rüdiger W. Veh, Misty J. Eaton, Serguei N. Skatchkov

**Affiliations:** 1 Department of Physiology, Universidad Central del Caribe, Bayamón, PR 00956, USA; 2 Department of Biochemistry, Universidad Central del Caribe, Bayamón, PR 00956, USA; 3 Department of Chiropractic, Universidad Central del Caribe, Bayamón, PR 00956, USA; 4 Department of Natural Sciences, University of Puerto Rico, Aguadilla, PR 00603, USA; 5 Department of Cell Biology, Washington University School of Medicine, St. Louis, MO 63110, USA; 6 Institut für Zell- und Neurobiologie, Charité, 10115 Berlin, Germany

**Keywords:** polyamines, glial syncytium, astrocytes, connexin

## Abstract

The interest in astrocytes, the silent brain cells that accumulate polyamines (PAs), is growing. PAs exert anti-inflammatory, antioxidant, antidepressant, neuroprotective, and other beneficial effects, including increasing longevity in vivo. Unlike neurons, astrocytes are extensively coupled to others via connexin (Cx) gap junctions (GJs). Although there are striking modulatory effects of PAs on neuronal receptors and channels, PA regulation of the astrocytic GJs is not well understood. We studied GJ-propagation using molecules of different (i) electrical charge, (ii) structure, and (iii) molecular weight. Loading single astrocytes with patch pipettes containing membrane-impermeable dyes, we observed that (i) even small molecules do not easily permeate astrocytic GJs, (ii) the ratio of the charge to weight of these molecules is the key determinant of GJ permeation, (iii) the PA spermine (SPM) induced the propagation of negatively charged molecules via GJs, (iv) while no effects were observed on propagation of macromolecules with net-zero charge. The GJ uncoupler carbenoxolone (CBX) blocked such propagation. Taken together, these findings indicate that SPM is essential for astrocytic GJ communication and selectively facilitates intracellular propagation via GJs for negatively charged molecules through glial syncytium.

## 1. Introduction

In brain, neurons and glial cells are isolated one from another by the aquatic-intercellular space. Astrocytes are connected by large diameter connexin (Cx) gap junction pores (GJs) that facilitate astrocytic communication and function [1,2,3,4]. Over the past decade, the interest about astrocytes [5,6,7,8] and polyamines (PAs) in the nervous system [9,10,11,12,13,14,15,16,17,18] has increased. Astrocytes have extensive communication via Cx GJ channels that make the three-dimensional astrocytic syncytium large, electrically isopotential [4,19], and unique [10,11,19,20]. Furthermore, astrocytes, but not neurons, accumulate PAs [15,18,21,22,23] and provide PA fluxes via Cx GJs [24], specifically via Cx43 GJs [25,26]. Half-GJs, Cx43-hemichannels (HCs) are open to the extracellular space and are pathways for PA release from astrocytes [14] as well as for glutamate [27], glutathione [28], D-serine [29], and ATP [30]. Metabolites are transferred via glial Cx43 and Cx30 GJs for large distances where they may affect the neuronal environment [2,20,29,31,32,33]. Therefore, GJs and HCs together with transporters like SLC18B1 [34] and SLC22A [11,15,16,35,36,37] are PA uptake/release pathways in astrocytes and other glial cells. 

Recently, PAs and their derivatives have been recognized as novel gliotransmitters [10,11,14,18,24,34]. Acetylated PAs [38], acrolein, putreanine, and hypusine [15] are novel (patho)physiological-glial PA derivatives and markers. Intriguingly, the PAs spermidine (SPD) and spermine (SPM) are not synthesized in adult astrocytes [9,39,40], but are accumulated in these glia, suggesting a high rate of unidirectional PA uptake in astrocytes [14,34,41]. Therefore, such PA accumulation is clearly evolutionarily determined; it is found throughout the brain [21,42], retina [22,23,43], peripheral nervous system [44], and in glial-neuronal co-cultures [45] of multiple species, including humans [22]. While the brain contains high amounts of SPD and SPM, the smaller PA putrescine is present at much lower concentrations, about 2% of the total PA content [46,47,48]. Conversion of putrescine to GABA in astrocytes was suggested [3,49,50] and, indeed, astrocytes release GABA [15,18]. GABA released from astrocytes confers tonic inhibition and is an anti-epileptic glial agent [18,49].

SPD/SPM are observed only in a few adult neurons and neurosecretory-neuronal- synaptic terminals [42] and the mechanisms of PA accumulation in glia, action, and astrocyte-to-astrocyte communication remain a mystery [14,15,24,41]. PAs underlie many glial cell-involved CNS diseases and syndromes [3,10,11,15]. PA catabolism in brain is regulated by Spermidine/Spermine N 1-Acetyltransferase (SSAT), which catalyzes acetylation of SPD and SPM and polyamine oxidases (PAO, SMOX); these catalyze the oxidation of acetylated PAs to dangerous radicals, such as hydrogen peroxide and propyl aldehyde (3-aminopropanal) causing excitotoxicity, epilepsy, seizure, and astrogliosis [16,17,51,52]. There are ways to quench oxidation of PAs and to induce neuroprotection by blocking DAO, PAO, and SMOX by aminoguanidine, chloroquine [53,54], and probably by SPD which has a clear neuroprotective effect [55]. Both SPD and D-glucosamine are found to promote longevity [56].

Recently, it was shown that glial cell-to-cell communication in brain [24], retina [57], and specifically in cells expressing Cx43-GJs [26] is strongly dependent on the intracellular store of PAs. PAs enter Cx43-expressing cells via PA uptake from the extracellular space and then act internally to affect cell-to-cell communication [58]. We have previously shown that intracellular SPM and SPD open Cx43-GJs [25,26]. The opening of GJs is crucial for processes, such as glucose transport [59], potassium buffering [4,60,61], and large-scale distribution of energetic substrates and signaling molecules throughout the syncytium [2,61,62,63,64].

Intriguingly, the trafficking of glucose molecules with zero charge (fluorescent 2-NBDG and fluorescent 6-NBGD) via GJs was about two orders higher in the astrocytic syncytium, compared with a negatively charged phosphorylated glucose, such as 2-NBGD-6P (glucose-6-phosphate) [31,59]. Therefore, this unexpected difference in charge-dependent trafficking of molecules through astrocytic GJs prompted us to investigate (i) the propagation of differently charged molecules with (ii) varying molecular weights, and (iii) the role the PA SPM may play. We have previously shown that SPM (1) opens gap junctions in astrocytes [24] and (2) in Novikoff cells [26] which natively express Cx43, where SPM (3) removes hydrogen block [25] and (4) calcium block of Cx43 [26]. A part of the current work was presented in abstract form at the Annual Society for Neuroscience meeting [65].

## 2. Materials and Methods

### 2.1. Animals

All procedures were carried out in accordance with the National Institute of Health guidelines for the humane treatment of laboratory animals and with the approval from the Universidad Central del Caribe Institutional Animal Care and Use Committee.

### 2.2. Brain Slice Preparation

To avoid age-dependent variation in Cx-based communication, we used brains of 25–30 postnatal-day-old Sprague-Dawley (P25–P30) rats. At this age, glial cells are mature and both Cx 43 gap junctions and Kir4.1 channels are fully developed [3,66,67,68]. Transverse 350 µm thick hippocampal slices were prepared from the brains of Sprague-Dawley rats of both sexes. Brains were dissected in ice-cold artificial-cerebrospinal fluid (ACSF) saturated with 5% CO_2_/95% O_2_ and slices were cut using a vibratome (VT1000S; Leica, Nussloch, Germany). The slices were then incubated for recovery in a standard ACSF solution containing (mM) 127 NaCl, 2.5 KCl, 1 MgCl_2_, 2 CaCl_2_, 1.25 NaH_2_PO_4_, 10 glucose, 26 NaHCO_3_, gassed with 5% CO_2_/95% O_2_, pH 7.4, (osmolarity 305 mOsm/L). The incubation was for 20 min at 35 °C and an additional 10 min at RT. After 30 min of total incubation, slices were placed in a recording chamber (0.5 mL volume) and superfused continuously with oxygenated ACSF at room temperature (23–24 °C, 1 mL/min). Whole cell recording and fluorescent dye tracing studies were performed as described previously [24,57,65].

### 2.3. Electrophysiology

MX7500R/L manipulators with MC-1000 drives (Siskiyou Inc., Grants Pass, OR, USA) were used for positioning micropipettes for whole-cell voltage-clamp and current-clamp recordings. Astrocytes were clamped with patch pipettes made from borosilicate-glass tubing (OD 1.5 mm, ID 1.0 mm; World Precision Instruments, Sarasota, FL, USA) pulled in four steps using a Flaming-Brown P-97 pipette puller (Sutter Instruments Corporation, Novato, CA, USA). Pipettes were filled with intracellular solution (ICS) containing (mM) 117 K-gluconate, 13 KCl, 2 MgCl_2_, 10 HEPES, (1 SPM-Cl was used in part of experiments), pH adjusted to 7.2 with KOH (osmolarity ~285 mOsm/L). After filling with ICS, the final micropipette resistance was close to 8 MΩ, which was optimized for astrocyte recordings to achieve seals of more than 3 GΩ on cell membranes. In slices, voltage clamping and current recording in the whole-cell patch-clamp mode from astrocytes were performed using MultiClamp700B (Molecular Devices, San Jose, CA, USA). The pClamp 10 software package (Molecular Devices Inc., San Jose, CA, USA) was used for data acquisition and analysis. The traces were low-pass filtered at 3 kHz and digitized at 10 kHz (Axon DigiData 1440A interface) (Molecular Devices Inc., San Jose, CA, USA). The electrophysiological data were analyzed with the software, Clampfit 10.2 (Molecular Devices, San Jose, CA, USA).

Astrocytes were dialyzed in whole-cell configuration in current clamp (zero-current) mode. Each single astrocyte tested was loaded via a patch pipette with the physiological ICS containing different fluorescent dyes in the presence or absence of 1 mM SPM. This concentration of SPM was chosen because it is close to the cytoplasmic SPM concentration of about 800 µM in glia [69].

Astrocyte recordings were accepted only if (i) the membrane potential was negative to ~−70 mV and (ii) if cells had a linear current-voltage relation (adult-passive astrocytes) and (iii) low input resistance (<20 MΩ). We used voltage-clamp mode to test current-to-voltage relationships and to characterize the type of cells. The holding potential was kept equal to the resting potential (to keep zero current), and cells were stimulated by short 60 ms voltage steps applied from the holding potential to −150, and then up to +150 mV in 10 mV increments, or by a “step-ramp” protocol using a step to −120 mV (for 100 ms), followed by a rising voltage ramp to +100 mV during 80 ms, and then a step back to resting voltage. We selected short protocols with a well-tolerated voltage range to help keep cells healthy while cell type was determined. Passive astrocytes, which are part of a large syncytium, produced linear I/V curves and were further utilized for GJ communication experiments. We avoided using astrocytes localized closer to the slice surface because those astrocytes may be artificially decoupled by the trauma obtained during cutting the slice. Instead, we recorded only from astrocytes situated at least 100 µm below the surface of the slice [70]. Therefore, the data were collected from well-coupled mature-passive astrocytes in the syncytium [66] with isopotentiality [19], but not from other cell types.

### 2.4. Cell Selection and Morphology

**Figure 1:**
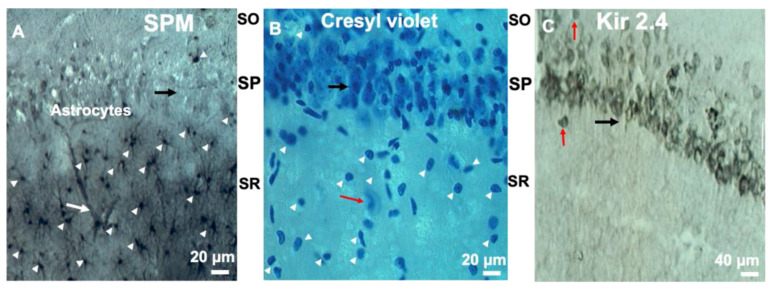
Localization of SPM in astrocytes and Kir 2.4 in neurons in CA1 hippocampus. (**A**) Immunocytochemical visualization of spermine/spermidine (SPM/SPD) accumulation in astrocytes (white arrowheads). Black arrow indicates the pyramidal cell area showing that pyramidal cells are not accumulating SPM and stay transparent. White arrow points to a blood vessel enwrapped by fine-astrocytic processes. (**B**) Staining with cresyl violet shows all cells (neurons and astrocytes) in the CA1 area of hippocampus. White arrowheads show small cells, likely astrocytes. Red arrow points to large interneuron. (**C**) Red arrows show interneurons, whereas black arrows show pyramidal cells. The neuronal channel Kir2.4 is localized in neurons. SO, SP, and SR are stratum oriens, pyramidale, and radiatum, respectively. Note: most of the patch-clamp recordings ignore the fact that the astrocytic cytoplasm contains SPM.

Morphologically and electrophysiologically identified astrocytes were used but not neurons, NG-2 cells, and oligodendrocytes. Astrocytes accumulating SPM/SPD, pyramidal neurons, and interneurons show different localizations (Figure 1) in stratum pyramidale (SP), in stratum radiatum (SR), and in stratum oriens (SO). In living brain slices, the astrocytes were visualized and identified using several procedures: (i) by their localization in SR and SO, (ii) by their small size and (iii) by their passive electrical properties. We used an Olympus infrared microscope (BX51WI; Olympus, Shinjuku-ku, Tokyo, Japan) equipped with a 40X water-immersion objective and two cameras: a CCD-video-camera (XC-73, Sony, Tokyo, Japan) for infrared differential interference contrast (IR-DIC) with DIC optics; a second camera (DP30BW digital, Olympus, Shinjuku-ku, Tokyo, Japan) for fluorescent images with. DP controller software (Olympus, version 3.3.1.292) was used to visualize, record black and white images, and quantify the spread of the dyes.

We specifically selected the CA1 area of hippocampus where (i) astrocytes are clearly separated from pyramidal neurons in specific areas (which is not like in cortex where astrocytes are mixed with different neuronal cells) and (ii) the astrocyte size is about twice smaller than neurons (Figure 1). In addition, we tested electrophysiological properties of patched astrocytes. No neurons or NG2 cells were used. The cells patched have no spontaneous-synaptic potentials (either depolarizing or hyperpolarizing miniature potentials) and no spikes.

We visualized the dye-coupled astrocytes (loaded with membrane-impermeable dyes via the micropipette) and determined the size of the syncytium, the heterogeneous connectivity, and the extent of dye propagation in recipient astrocytes after 10 min of single-cell dialysis. Counting of coupled cells was carried out in a single X-Y plane (focused on the cell which was filled with fluorescent dye) and was equally applied for all the slices and procedures tested. In addition, for high-resolution imaging and for retention of the dyes after 10 min of single-cell dialysis, each slice was immersed in freshly prepared 4% paraformaldehyde in 0.1 M PBS solution, pH 7.4, in 5 mL tubes. After shaking for 5 min at room temperature, the slices were stored at 4 °C for 1 h. Freshly fixed slices were then processed via confocal microscopy to obtain fresh additional 3D images showing propagation of different dyes with and without cytoplasmic SPM in the glial syncytium using a confocal-scanning microscope (LSM 510 META, Zeiss, Oberkochen, Germany and Olympus Fluoview FV1200, Olympus, Japan).

### 2.5. Immunohistochemistry

**Figure 2:**
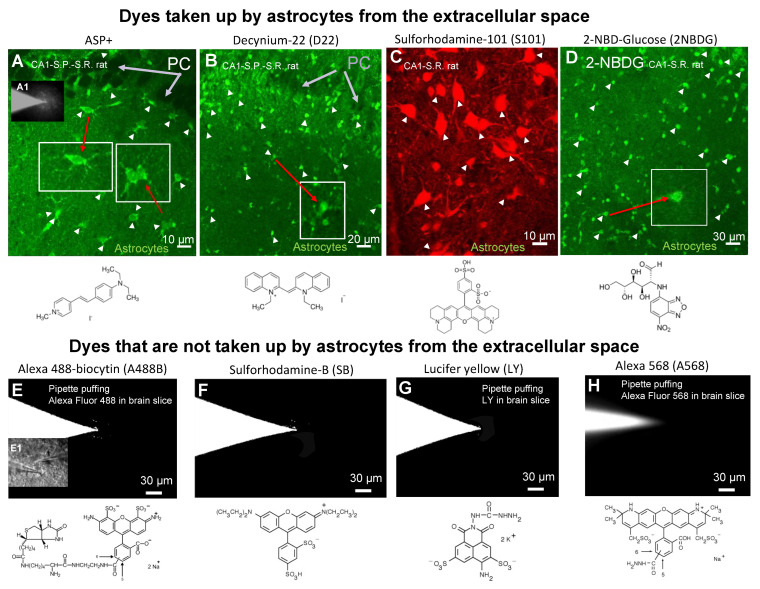
Dyes taken up versus membrane-impermeable dyes in astroglial syncytium. (**A**–**D**): molecules which are taken up by astrocytes (white arrowheads and red arrows), but by neurons (gray arrows) and (**E**–**H**): molecules which were not taken up by astrocytes in brain slices. (**A**): 4-(4-(dimethylamino)-styryl)-*N*-methylpyridinium (ASP+), MW = 366.24, net charge = +1; (**B**): 1,1′-Diethyl-2,2′-cyanine iodide or decynium22 (D22), MW = 454.35, charge = +1; (**C**): Sulforhodamine-101 (S101), MW = 606.71, net charge = 0; (**D**): 2-NBD-Glucose (2NBDG), MW = 342.26, net charge = 0; (**E**): Alexa 488-biocytin (A488B), MW = 974.98, net charge = −2; (**E1**) Brightfield image showing patched astrocyte. (**F**): Sulforhodamine B (SB), MW = 558.66, net charge = 0; (**G**): Lucifer yellow (LY), MW = 521.57, net charge = −2. (**H**): Alexa 568 (A568), MW = 730.74, net charge = −1.

**Figure 3:**
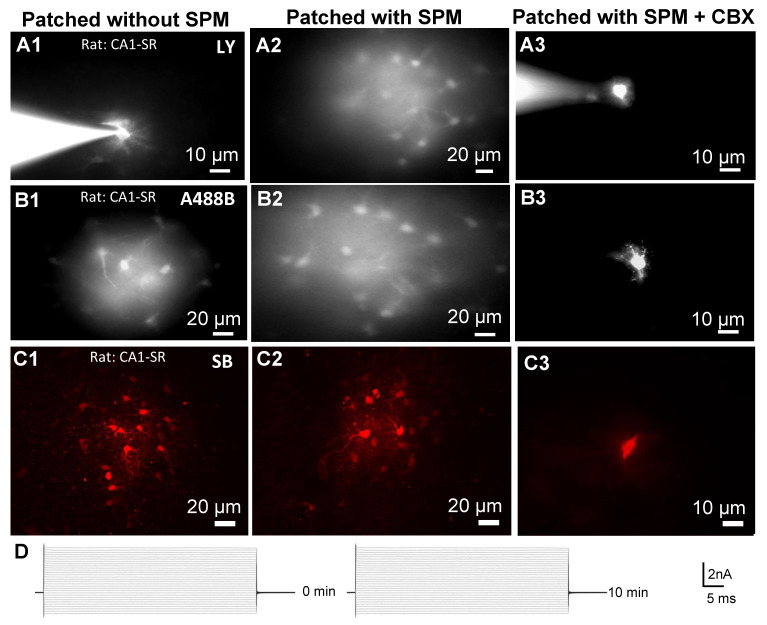
Confocal images of Lucifer Yellow (LY), Alexa 488-biocytin (A488B) and Sulforhodamine-B (SB) propagation in the astrocytic syncytium of adult rat CA1 hippocampus. (**A1**–**A3**): shows LY (1 mM) dye propagation in the astrocyte syncytium without SPM (**A1**), with 1 mM SPM (**A2**) and with 1 mM SPM after 20 min pretreatment with 200 µM CBX (**A3**). The pipette containing LY patching a single astrocyte is visible on the left side of A1 and A3. (**B1**–**B3**): shows A488B (100 µM) dye propagation without SPM (**B1**), with 1 mM SPM (**B2**) and with 1 mM SPM after 20 min pretreatment with 200 µM CBX (**B3**). (**C1**–**C3**): shows SB (2 mM) dye propagation in the astrocyte syncytium without SPM (**C1**), with 1 mM SPM (**C2**) and with 1 mM SPM after 20 min pretreatment with 200 µM CBX (**C3**). (**D**): Astrocyte currents were measured at the beginning (left) and at the end (right) of experiments. Responses to voltage steps applied from the holding potential in the range from −150 mV to +150 mV with 10 mV increments during 60 ms are shown. The linear conductances represent mature-passive astrocytes and they are stable during the 10 min of the experiment.

**Figure 4:**
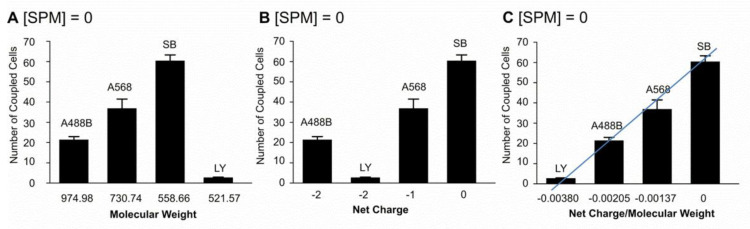
Comparison of astrocyte-to-astrocyte coupling in the control group (in the absence of spermine ([SPM] = 0 in the intracellular solution)) and their dependence on the characteristics of the individual dyes. (**A**–**C**): The number of coupled cells in the Alexa 488-biocytin group (A488B, 21.4 ± 1.6, N = 11, Vm = −79.7 ± 1.4 mV), Alexa 568 (A568, 36.9 ± 4.6 cells; Vm = −80 ± 0.8 mV, N = 10), Sulforhodamine B (SB, 60.4 ± 2.9, N = 7, Vm = −79.4 ± 1.1 mV) and Lucifer Yellow (LY, 2.7 ± 0.2, N = 15, Vm = −80.2 ± 0.7 mV) is dependent on (**A**) molecular weight of the dye, (**B**) net charge of the dye and correlates (**C**) with the ratio of net charge and molecular weight of the dye. Error bars indicate standard error of mean (SEM).

**Figure 5:**
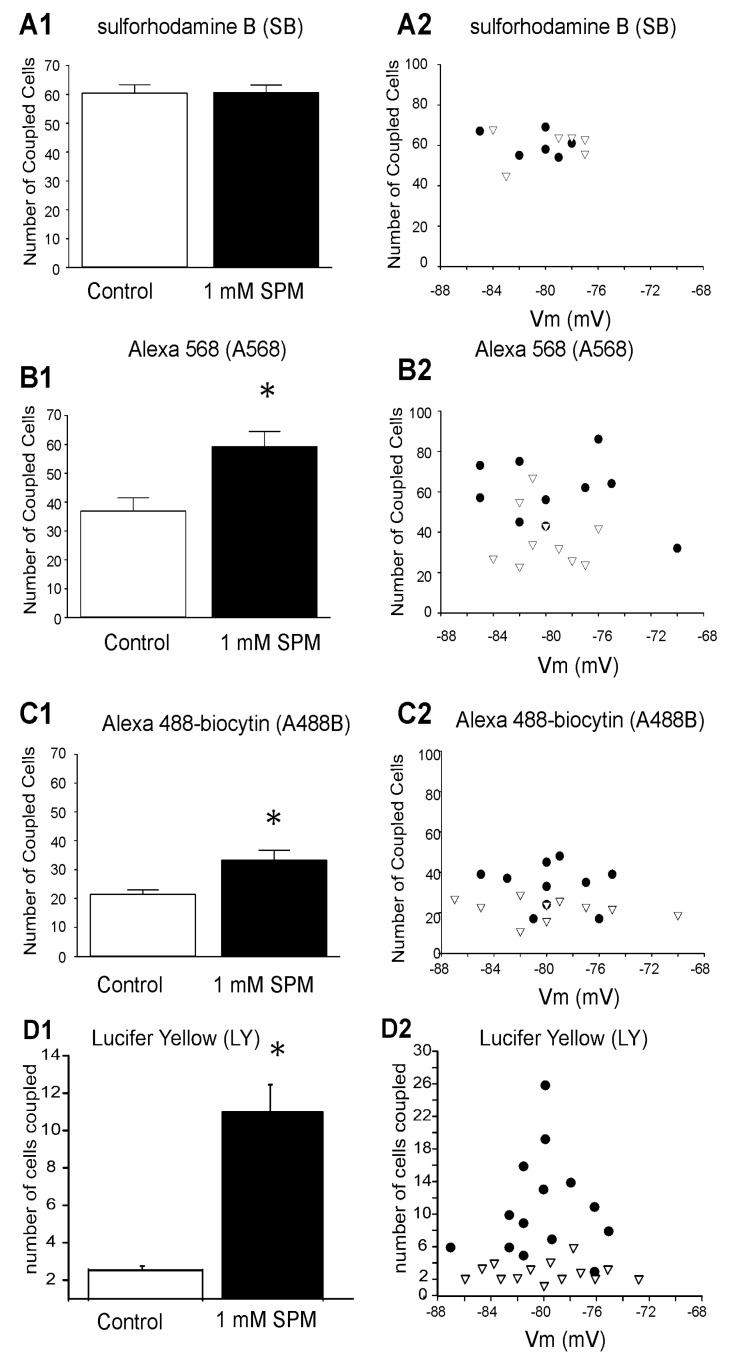
Effect of intracellular spermine (SPM) on propagation of electrically neutral and charged dyes through the astroglial syncytium. (**A1**) Using neutral-polar dye SB, electrically neutral amine Sulforhodamine B (2 mM), 1 mM spermine (SPM) included in the pipette has no effect on the amount of coupling cells: control (60.4 ± 2.9, N = 6) compared to the SPM-treated group (60.7 ± 2.5, N = 6). (**B1**) Using negatively charged dye A568, Alexa 568 (200 µM), 1 mM spermine increases the extent of coupling, control (36.9 ± 4.6, N = 10) compared to the SPM-treated group (59.3 ± 5.2, N = 10). (**C1**) 1 mM spermine increases the amount of coupling of cells filled with negatively charged Alexa 488-biocytin (100 µM), control (21.4 ± 1.6, N = 11) compared to the SPM-treated group (33.3 ± 3.4, N = 10). The asterisks indicate a significant difference between the control and SPM-treated groups. (**D1**) 1 mM SPM strongly increases the amount of coupling of cells filled with negatively charged LY, Lucifer Yellow (100 µM), control (3.5 ± 1.7, N = 15) compared to SPM-treated group (11.5 ± 3.6, N = 14). The asterisks indicate a significant difference between the control and SPM-treated groups (*p* < 0.01), error bars indicate standard error of mean (SEM). (**A2**–**D2**): There is no correlation found between coupling and the astrocytic-membrane potential in the control group (white triangles) or in the spermine-treated group (black-filled circles).

**Figure 6:**
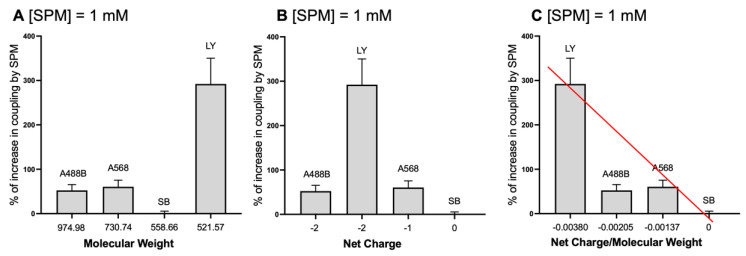
Spermine strongly increases propagation of negatively charged molecules: Dependence of the % increase in astrocytic coupling by SPM (1 mM) on the characteristics of the individual dyes. SPM-induced a 56% increase in the Alexa 488-biocytin group, a 61% increase in the Alexa 568 group, no change in the Sulforhodamine B group and a 292% increase in the Lucifer Yellow group. These changes were dependent on (**A**) molecular weight of the dye, (**B**) net charge of the dye and correlate (**C**) with the ratio of net charge and molecular weight of the dye. The red line shows the strength of SPM to increase propagation of various negatively charged molecules through GJs in the astrocytic syncytium. Error bars indicate standard error of the mean (SEM). Note: compare with Figure 4 where endogenous PAs were dialyzed out of the astrocytes.

Brain slices (350 µm thick from 25–30 days old Sprague Dawley rats) were used for immunohistochemical studies. We used a total of 56 rats for the experiments presented in Figure 1, Figure 2, Figure 3, Figure 4, Figure 5 and Figure 6. From each rat, we obtained 5 brain slices. From each brain slice, we used one to two astrocytes to test-dye propagation. The slices (prepared as described above) were fixed in a solution of 4% paraformaldehyde, 0.05% glutaraldehyde, and 0.2% picric acid in 0.1 M phosphate buffer, pH 7.4, for 16 h. After fixation, the slices were cryoprotected by immersion in 0.15 M sucrose in 0.1 M phosphate buffer, pH 7.4 (for 24 h), 0.5 M sucrose (for 24 h), and 0.8 M sucrose (for 48 h). Subsequently, they were frozen at −60 °C in liquid pentane and then stored in a −80 °C freezer until next use. The 350 µm slices were mounted on a platform of frozen OCT compound in a cryostat and resectioned at 25 µm thickness using Leica CM1520 cryostat (Leica Biosystems, Wetzlar, Germany) at −20 °C. The sections were pretreated with 1% sodium borohydride in PBS for 15 min, and subsequently permeabilized with 0.3% Triton X-100 for 30 min. The primary anti-SPM and anti-Kir2.4 antibodies were generated and characterized in the laboratory of Dr. Rüdiger Veh [21,22,71]. Antibodies were used at dilutions of anti-SPM at 1:300 and anti-Kir2.4 at 1:5000. After incubation with the primary antibody for 36 h at 4 °C, freely floating sections were treated with the secondary antibody (biotinylated goat anti-rabbit IgG 1:2000, Vector Laboratories, Inc., Newark, CA, USA) for 18 h, and then with an ABC complex (Vectastain Elite, 1:1000, Vector Laboratory) for 6 h. Peroxidase activity was revealed with 1.4 mM 3,3-diaminobenzidine (DAB), 10 mM imidazole, 0.3% nickel ammonium sulfate and 0.015% hydrogen peroxide in 50 mM Tris-HCl, pH 7.6 for 3 min at room temperature. Controls were obtained by omitting the primary antibody and occasionally supplemented by cell-body staining (Cresyl violet). All sections were developed for 3 min, mounted on glasses and dehydrated through a graded series of ethanol, transferred into xylene and coverslipped with Entellan (Merck, Darmstadt, Germany).

### 2.6. Materials

In this study, we used several different charged (negative, positive, and neutral) molecules of fluorescent dyes. Alexa-488-biocytin, Alexa-568, and sulforhodamine-101 were purchased from Molecular Probes. Lucifer Yellow, sulforhodamine-B, 1,1-Diethyl-2,2-cyanine iodide (decynium22, D22) 2-NBD-glucose, and 4-(4-(dimethylamino)-styryl)-N-methylpyridinium, (ASP+) were obtained from Sigma Chemical Co., Ltd. (St. Louis, MO, USA). For testing the compounds, one of these negatively or positively charged, or polar molecules, was added to the ICS at the following concentrations: 2 mg/mL (~3.6 µM) 2-NBD-Glucose [59]; 2 µM ASP+ [72]; 1 µM D-22 [72,73]; 1 µM sulforhodamine-101 [74,75,76]; 100 µM Alexa 488-biocytin [65]; 1 mM Lucifer Yellow [24,77]; 200 µM Alexa 568 [65]; and 2 mM sulforhodamine-B [57].

Carbenoxolone (200 µM CBX), a gap junction uncoupler, used in this study to block fluorescent dye propagation was purchased from Sigma Chemical Co., Ltd. (St. Louis, MO, USA).

### 2.7. Data Analysis

Data were analyzed using pCLAMP-10, version 10.4.0.36 (Molecular Devices, San Jose, CA, USA) or Origin 8 software, version 8.0725 (OriginLab, Northampton, MA, USA) and were reported as mean ± SEM. Significant differences between groups of data were evaluated using the paired Student’s *t*-test. Statistical significance was expected if *p* < 0.05. Note: brain slices incubated for longer than 1 h were not used. In addition, all the data were tested for normality using the Shapiro-Wilk test (they passed the normality test).

## 3. Results

### 3.1. Localization of Polyamines Spermine and Spermidine (SPM/SPD) in Astrocytes and Polyamine-Sensitive Potassium Inwardly Rectifying Channels Kir2.4 in Neurons

To visualize astrocytes versus neurons, we used antibodies against the PAs SPD/SPM and the neuronal Kir2.4 channels. We found localization of PAs in astrocytes (Figure 1A, white arrowheads) while Kir2.4 channels are localized in pyramidal cells (Figure 1C, black arrow) and in interneurons (Figure 1C, red arrow), but not in glia (Figure 1C). The Kir2.x family displays the highest PA-sensitivity and SPM regulates the permeability of these channels [78]. PAs were found in glia and astrocytes are co-localized with interneurons (Figure 1). From the literature, it is known that the interneurons and astrocytes express PA sensitive AMPAR and KAR (AMPA-kainate receptors) [79,80,81,82,83,84], n-n-AChR [85,86], NMDAR [87,88], TRP channels [89,90,91], and a variety of Kir channels [69,78,92]. This suggests that glial cells can use SPM and SPD to regulate not only their own Kir4.1 channels [22,23,69] or Cx43 GJs [26,93], but also the neighboring neuronal receptors and channels by SPM, if released. The astrocytes may also propagate PAs through the astrocytic syncytium via GJs [24] and then release them into the neuronal network under special conditions, such as gliotoxin [18] or during uptake/release [14].

### 3.2. Determination of Membrane Permeable (Uptake) versus Impermeable Dyes to Study Astrocyte-to-Astrocyte Dye Propagation

The next question was: which molecules may be used to analyze transfer via astrocytic GJs? For this, we need to distinguish between membrane-permeable versus membrane-impermeable dyes. To visualize the diffusion only through the gap-junctions, we carefully selected the dyes we would use in our experiments. We found that some dyes were crossing the astrocytic membrane and, therefore, were not suitable to study intracellular propagation through the astrocytic syncytium.

Several dyes (Figure 2) were taken up by the astrocytes during the time when the puffing tip of patch micropipette was approaching the cells, and therefore, were not suitable for the present study. 4-(4-dimethylaminostyryl)-N-methylpyridinium (ASP+), sulforhodamine-101 (S101), and decynium-22 (D22) were rapidly, robustly, and selectively taken up by astrocytes, but not neurons (Figure 2A–C). Similarly, 2-NBDG was taken up by astrocytes (Figure 2D). Our previous data showed that membrane uptake of amines could occur in astrocytes and gliomas [72,94] via SLC22A-type transporters that also transport ASP+, MPP+ (amines) and PAs [36]. Therefore, amino group-containing dyes could be substrates for polyspecific mono- and polyamine transporters [36,95] expressed in glia [5,34,35,96]. In general, positively charged dyes with cationic-functional groups, such as −NR3+ or =NR2+ (such as, ASP+, MPP+, and thiazine, D22) were deemed not suitable for the present study. Also, fluorescent glucose (Figure 2D) was taken up by astrocytes most probably by the GLUT1 transporter [97,98]. It would be difficult to determine if the dyes shown in Figure 2A–D were taken up by the cells through the membrane or propagated to neighboring cells via GJs communication; therefore, these dyes were not suitable for further experiments for the intracellular propagation study.

In addition, there are large pores, such as connexin [1,64,99,100] and pannexin hemichannels [2], which can potentially take up the fluorescent molecules from the extracellular space, particularly if astrocytes are in a low metabolic stage or during gliosis [99,101]. To minimize this uptake, we prevented activation and opening of large pores, such as connexin and pannexin hemichannels, P2X receptors, and others by (i) keeping concentrations of extracellular divalent cations at high physiological millimolar concentrations (block of Cx43 HCs), (ii) avoiding metabolic deficiency, and (iii) avoiding the use of any agonists which open pannexin hemichannels and P2X receptors. Figure 2E–H shows compounds that are not taken up from the extracellular spaces (i.e., membrane impermeable). These molecules include Alexa 488-biocytin (A488B), sulforhodamine B (SB), Lucifer yellow (LY), and Alexa 568 (A568), and were used for the dye-propagation studies described below.

### 3.3. Astrocyte-to-Astrocyte Dye Spreading in Absence and Presence of Polyamines in Glia

Using the whole-cell configuration, we examined the propagation of various dyes through the astrocytic syncytium with and without SPM in the patch pipette. We used a concentration of SPM (1 mM) close to the physiological concentration [69] to estimate the physiological effect of SPM on molecular propagation in the glial syncytium. We kept the holding potential equal to membrane potential to maintain iosopotentiality [19]. Isopotentiality means that astrocytes “clamp” each other electrically via gap junctions providing equal conditions where each astrocyte in this glial network has the same membrane potential as coupled neighbors. It is important because changes of the trans-junctional potential (trans-junctional voltage gradient, GJ gradient) may block electrical currents from cell-to-cell [102,103] and specifically can block dye permeation because propagation is dependent on the difference between the membrane potentials of GJ coupled cells [103,104]. Also, keeping the holding potential equal to resting minimizes unwanted ionic currents via membranes, and avoids ionic and molecular transport across the surface membrane. The latter can prevent calcium load and minimize changes of cytoplasmic calcium to which GJs are extremely sensitive [26]. Finally, since magnesium [105] and hydrogen gradients [25,106] play a role for gap junction opening/closing, we kept these concentrations normal, stable, and equal for each astrocyte patched. These conditions were equally applied in each experiment and for each cell to keep the protocol consistent.

To obtain representative images (Figure 3) of dye propagation in stratum radiatum of rat CA1 hippocampus, the soma of a single astrocyte was clamped in whole-cell mode and the cell was dialyzed using either SPM-free or 1 mM SPM-containing ICS with 200 µM Alexa 568, 100 µM Alexa 488-biocytin, 2 mM SB, or 1 mM LY. These concentrations were close to those reported in the literature [77,107,108]. We compared differently charged dyes: two negatively charged dyes (−2 charge) but with largely different molecular weight: (i) Alexa 488-biocytin (A488B), (MW = 974.98, charge −2) and (ii) Lucifer Yellow (LY), (MW = 521.57, charge −2). Then, we used negatively charged dye (−1 charge) (iii) Alexa 568 (A568), (MW = 730.74 net charge = −1) and a polar molecule with net charge of zero (iv) Sulforhodamine B (SB), (MW = 558.66 with net charge = 0). These molecules were tested in terms of their propagation throughout the astrocytic syncytium in the presence and absence of SPM. We conducted the experiments in hippocampal brain slices under constant slice perfusion of 1 mL/min with ACSF. After achieving a gigaseal and cell opening, cells were clamped at zero current (at resting Em) in voltage-clamp mode, and i/V-test was made. Then the cells were dialyzed for 10 min. After 10 min, the pipette was carefully withdrawn away from the cell and fluorescent micrographs were obtained to quantify the number of coupled cells (see Section 2).

In the absence of SPM ([SPM] = 0, Figure 3(A1,B1,C1)), LY propagated only between few (or none) astrocytes (Figure 3(A1)), while 10–14 astrocytes (rarely up to 27 cells) were typically filled with LY when SPM was included in the pipette (Figure 3(A2)). To establish complete uncoupling of gap junctions, slices were perfused with carbenoxolone (200 µM), a gap junction uncoupler, for 20 min before penetration with a patch pipette containing the fluorescent dye. This procedure effectively blocked all dye propagation (Figure 3(A3)). Alexa 488-biocytin propagated relatively freely (Figure 3(B1)) and, in the presence of SPM, the number of coupled cells nearly doubled (Figure 3(B2)), but again, propagation was blocked by 200 µM CBX (Figure 3(B3)). Similar results were obtained for Alexa 568 (see Figure 4 and Figure 5). In contrast, propagation of SB was unaffected by SPM (Figure 3(C1,C2)), but SB propagation was also blocked by CBX justifying a GJ pathway (Figure 3(C3)). To monitor integrity of the cells, the membrane potential and current-voltage relationship were obtained twice: immediately after breaking into whole-cell mode and a second time after 10 min of cell dialysis (Figure 3D). Only cells that showed stable electrophysiological properties as passive astrocytes [4,19] were included in the study. Figure 3D shows an example of a current recording from a passive astrocyte (with linear I/V-curve) and such astrocytes were used for further analysis.

### 3.4. Extent of Dye-Spreading between Astrocytes Located in Stratum Radiatum of Rat CA1 Hippocampus Is Charge- and MW-Dependent and Correlates with the Ratio of Charge to MW: Polyamine-Independent Propagation in the Glial Syncytium

For statistical evaluation of astrocytic coupling, we used different slices from different animals for all fluorescent dyes used. Intuitively, the smaller molecular weight compound should permeate easier, faster, and for more distance than large dyes. Intriguingly and in contradiction to the obvious expectation, without SPM, LY (the lowest molecular weight dye) permeates via GJ syncytium much less than the larger molecular-weight dyes (Alexa 488-biocytin, Sulforhodamine B), indicating the opposite of what was predicted (Figure 4A). The average number of cells filled with LY was only 2.7 ± 0.2 (Figure 4A, Vm = −80.2 ± 0.7 mV, N = 15), whereas the average number of fluorescent cells filled by Alexa 488-biocytin was 21.4 ± 1.6 cells (Figure 4A, Vm = −79.7 ± 1.4 mV, N = 11). Alexa 568 spread to a greater number of coupled cells (36.9 ± 4.6 cells; Vm = −80 ± 0.8 mV, N = 10) and sulforhodamine B showed much more propagation with 60.4 ± 2.9 coupled cells (Vm = −79.4 ± 1.1 mV, N = 7). In general, A488B, A568, and SB that gradually differed by molecular weight showed the number of fluorescently labeled astrocytes correlated with the molecular weight of the fluorescent dye, with better molecular propagation of lower molecular-weight compounds, but not for LY (Figure 4A). Indeed, the substances with nearly equal molecular weights, such as LY and SB, have ~20 times different permeability (Figure 4A). The smallest molecule, Lucifer Yellow, was nearly 10 times less permeable than Alexa 488-biocytin which has the same −2 charge (Figure 4B). This suggests that the density of the charge per molecular weight (size) plays a role, such that when the negative charge is condensed as in the case of a small LY molecule, the permeability decreases. There was no clear dependence of the degree of dye-coupling on molecular weight alone (Figure 4A, note right column for LY) nor on net charge alone (Figure 4B (compare A488B with LY)).

Therefore, we calculated the ratio of the charge to weight of the molecules, and this appears to be a key determinant for molecular propagation. Indeed, the ratio of net charge and molecular weight of the dye clearly correlated with the number of cells coupled (Figure 4C). Therefore, we conclude that the interaction of dyes with the GJ pore can be dependent on both the electrical field of the pore and the density of the molecular charge of the dye. The next question was: does SPM change such behavior?

### 3.5. Spermine Differentially Affects Spreading of Electrically Negative and Neutral Dyes Independently on Membrane Potential

It is known that Cx pores may be negatively charged and retain charged molecules, as has been shown for Cx40 and Cx43 [109,110,111,112,113,114]. We, therefore, hypothesized that (i) the positively charged SPM (SPM+4 at neutral pH) may either neutralize the negative pore charge and relieve the electrical shield inside the GJ pores, or neutralize the negative charge of the trafficking molecules [109], and (ii) the membrane potential of the cells should not play a role since the astrocytes are joined in the isopotential syncytium [4,19], where the electrical profile of each GJ is identical. Therefore, the trans-junctional potential between communicating astrocytes is near zero and this is the best condition for GJ opening [103,106,109,110,111,112,113,114]. We tested this hypothesis. Recordings from control (no SPM) and SPM-filled cells were carried out in each slice in non-overlapping areas within 30 min of each other. In some experiments, controls were performed first, whereas in others, SPM treatment was carried out first. The number of coupled cells loaded with the electrically neutral dye SB (Sulforhodamine B, 2 mM) was 60.4 ± 2.9 (N = 7) for control, compared to 60.7 ± 2.5 (N = 6) for the SPM-treated group, indicating that inclusion of 1 mM SPM in the pipette did not alter the propagation of the neutral dye SB (Figure 5(A1)). On the other hand, 1 mM SPM significantly increased the extent of coupling of negatively charged Alexa 568 (Figure 5(B1)), control (36.9 ± 4.6, N = 10), compared to SPM-treated group (59.3 ± 5.2, N = 10, *p* < 0.01), and also increased the level of coupling of negatively charged Alexa 488-biocytin (Figure 5C1), control (21.4 ± 1.6, N = 11), compared to the SPM-treated group (33.3 ± 3.4, N = 10, *p* < 0.01). The most dramatic effect of SPM was seen with LY, control (2.7 ± 0.2, N = 15), compared to SPM-treated group (11.0 ± 1.4, N = 15; *p* < 0.0001) which was a 292% increase.

These results suggest that SPM affects only permeation of negatively charged molecules, while having no effect on neutral ones. Indeed, as was expected, we observed a lack of correlation between dye coupling and the astrocytic membrane potential in the control group and in the SPM-treated group (Figure 5(A2,B2,C2)). This is most probably due to well-known isopotentiality of the astrocytic syncytium where each coupled astrocyte has the same membrane potential as neighboring astrocytes [4,19]. For optimal GJ communication, the only major consideration is the equal trans-GJ membrane potential of neighboring cells [25,110,111,112,113,114]. This condition is present in the astrocytic syncytium [19].

### 3.6. Extent of Spermine Effect Correlates with the Ratio of Charge and MW of the Dyes

Using the above data (Figure 5), we calculated the % increase in cell coupling in response to SPM in the patch pipette, i.e., dialysis of the cytoplasm of the cell with SPM (Figure 5 and Figure 6) representing a percentile of SPM-induced increase of dye propagation (Figure 6A–C). SPM induced an increase in propagation of charged molecules of 56% for Alexa 488-biocytin (A488B) group, of 61% for Alexa 568 (A568) and of 292% for the smaller but strongly charged molecule of LY.

In contrast, there is no increase in Sulforhodamine B propagation (Figure 5A versus Figure 6A, SB). The dependence on the molecular weight of the dye (Figure 6A), net charge of the dye (Figure 6B), and the ratio of net charge to molecular weight of the dye (Figure 6C) is graphed. One important observation is that SPM increased equally permeation of A488 and A568; however, these molecules differed by molecular weight and charge (Figure 6A–C). That is not the case when SPM was absent in the cytoplasm (Figure 4C).

There is a clear dependence on the ratio of net charge to MW, indicating that charge density is the major factor in SPM-induced dye propagation.

## 4. Discussion

PAs play a key role in brain function and cell survival [12,34,40,47,115,116,117]. Recently, it was reviewed that PAs are anti-inflammatory, antioxidant, antithrombotic, antidepressant [10,11,13,15,16,118], and neuroprotective [55]. Indeed, both SPM and SPD have demonstrated similar effects. Both SPM and SPD are accumulated in healthy, but not in, damaged by gliotoxin astrocytes [18], and SPM is more effective than SPD and PUT as an opener of Cx43 [26]. For this reason, we chose to investigate the effect of SPM in the present study.

Also, PA content declines during aging [48] but SPD supplement is beneficial for memory [12,116,119,120,121], immune response [12], and increasing longevity [122]. Intriguingly, in the long-lived rodent, the naked-mole rat, PAs (in particular SPD, cadaverine, N8-acetyl-SPD, and N1,N8-diacetyl-SPD) were elevated, compared to its short-lived counterparts. Well known age-associated decline in SPD and N1-acetylspermidine levels in rodents did not occur in the naked-mole rat or was not even reversed (in the case of N1-acetylspermidine) [123]. It seems that PAs are key players in a large scope of diseases and age-related processes.

However, the functional analysis of astrocytic Cx43 GJs in respect to PAs is not well established. Interestingly, due to different amino-acid-pore sequences, Cx40 GJs are blocked by PAs [109,112,113], while Cx43 and Cx38 are not [25,26,109,124]. Glial cells do not have Cx38 and Cx40, but recently, robust glial Cx43 overexpression (over 6 times) was shown during aging-related tau astrogliopathy [125], making Cx43 and PAs of special interest. Cx43 is the dominant connexin subunit in cortical and hippocampal astrocytes accompanied by minor expression of Cx30 and Cx26 [20,100,118,126,127,128]. Consequently, cell-to-cell coupling for small ions and molecules is strongly reduced in Cx43 KO [58] and abolished in double-knockout mice lacking both Cx43 and Cx30 [32,59,61].

Intriguingly, studies in brain slices demonstrate variable cell-to-cell coupling [57,66,70,129,130], and large inconsistency has been reported in terms of the actual number of coupled astrocytes when using the fluorescent dyes in the same CA1 area of the hippocampus with and without SPM in the pipette (Figure 4, Figure 5 and Figure 6). Indeed, in the absence of SPM, very low coupling is typically reported [24,66,70,130]. As we can assume, astrocytes accumulate PAs in healthy brain (Figure 1A) which is consistent with an earlier study [15,21]. In contrast, (i) traumatic brain injury, (ii) rising acidity of the cytoplasm [131,132], and (iii) other conditions, such as gliotoxin [18], may lead to PA loss in astrocytes. The lowering pH results in blockade of gap junctions [105,106]; however, PAs restore GJ conductance by removing such hydrogen cation block of Cx43 [25,26]. Cells accumulate PAs [133], and SPM/SPD may be liberated from these stores and open Cx43 GJs by removing hydrogen and calcium-cation block [26]. Intriguingly, when SPM was not used, the trafficking of glucose with zero charge (fluorescent 2-NBDG or 6-NBGD) was robust in the astrocytic syncytium while the traffic of the phosphorylated-metabolite glucose-6-phosphate (2-NBGD-6P) was dramatically decreased by about 70% [31,59]. The phosphorylated glucose has a negative charge and is similar to negatively charged LY, A488, and A568 whose permeability is limited in the absence of SPM, but increased by SPM (Figure 4, Figure 5 and Figure 6). In addition, if LY is tagged to biocytin (which neutralizes a negative charge of LY), the astrocytic coupling was extensive and similar to zero charge sulforhodamine-B [59].

Since SPD synthesis is absent in adult astrocytes [39,134], the endogenous PAs of glial cells are typically washed out rapidly (during 70–120 s) after attainment of whole-cell configuration [69]. Astrocytes can lose SPM during brain-slice preparation and incubation (unpublished observations); therefore, inclusion of SPM intracellularly at a concentration shown to be physiologically present in vivo in glial cells [69] considerably increases propagation of negatively charged dyes, such as Alexa 568 and Alexa 488-biocytin, and most dramatically, the relatively small and highly charged LY (Figure 3, Figure 4, Figure 5 and Figure 6). We suggest that different results in the literature may be due to variation in PA content and factors, such as the species and age of the animal, area of the brain or retina, molecular weight, charge, size, and shape of the dye.

As we show, without SPM, none (Figure 3(A1)) or few astrocytes (Figure 5(D2)) showed LY propagation, which is in good agreement with previous work [24,70,130]. We infer that SPM itself must propagate through gap junctions because it opens LY spreading further than just the adjacent astrocyte and can cause robust coupling of cells in the distant part of the syncytium (Figure 3(A2)). Therefore, the native SPM concentration may be rapidly equilibrated among astrocytes through gap junctions and, thereby, help to keep the network chemically and electrically coupled. This may help to maintain the isopotentiality that was found previously [4,19].

PA signaling through gap junctions may be a key to regulate cell proliferation [109], particularly of astrocytes [41] and gap-junction organization in mammalian tissues [135]. Our finding suggests a mechanism by which SPM could facilitate diffusion of negatively charged molecules, without having an effect on neutral ones. These possibilities include: direct binding of the PA cations (SPM^4+^) to negatively charged dye molecules as was shown for acid proteins, ATP, and RNA, etc. in the cytoplasm [133]. By binding and neutralizing the negative charge of these molecules, PAs can enhance their permeation via GJ pores. It is known that astrocytes modulate activity of neurons by release of such negatively charged transmitters via hemichannels [1,64,99,136,137]; however, the studies did not highlight whether these molecules are bound to positively charged cations in the cytoplasm, such as PAs, that was shown [133]. So, an acid pH shift occurring in glial cells after their activation may lead to liberation of PAs from cytoplasmic buffers (with ATP, adenosine, proteins, phosphates, etc. [133]) and result in GJ regulation [25,26].

An alternative, but only theoretical explanation, could be that positively charged SPM^4+^ binds directly in the Cx43-pore and neutralizes the charge in this pore, and thus, making the pore wall electrically inert and enhancing the propagation of negatively charged molecules. Testing this mechanism in a separate large study is required. Therefore, PAs and their derivatives may serve many functions both within the astrocytic cytoplasm and upon being released from glial cells [10,11,14,15] to the extrasynaptic receptors localized in areas which are less studied.

## 5. Conclusions

In conclusion, our findings suggest that we may predict the extent of coupling between astrocytes for novel, untested molecules or fluorescent dyes by knowing MW and charge, but most importantly, for biologically active molecules and drugs. By extension, this suggests that SPM will facilitate diffusion of negatively charged intracellular-organic and -inorganic anions, amino acids, polypeptides, and other signaling molecules through the astrocytic syncytium, and may therefore, have profound effects on glial-neuronal signaling.

## Data Availability

Additional data supporting the conclusions of this finding will be made available without undue reservation.
